# CXCL10 is produced in hepatitis A virus-infected cells in an IRF3-dependent but IFN-independent manner

**DOI:** 10.1038/s41598-017-06784-x

**Published:** 2017-07-25

**Authors:** Pil Soo Sung, Seon-Hui Hong, Jeewon Lee, Su-Hyung Park, Seung Kew Yoon, Woo Jin Chung, Eui-Cheol Shin

**Affiliations:** 1 Laboratory of Immunology and Infectious Diseases, Graduate School of Medical Science and Engineering, KAIST, Daejeon, Republic of Korea; 2 Division of Hepatology, Department of Internal Medicine, Seoul St. Mary’s Hospital, The Catholic University of Korea, Seoul, Republic of Korea; 3 Biomedical Science and Engineering Interdisciplinary Program, KAIST, Daejeon, Republic of Korea; 4 Laboratory of Translational Immunology and Vaccinology, Graduate School of Medical Science and Engineering, KAIST, Daejeon, Republic of Korea; 5 Department of Internal Medicine, Keimyung University School of Medicine, Daegu, Republic of Korea

## Abstract

Acute hepatitis A caused by hepatitis A virus (HAV) infection is accompanied by severe liver injury in adult patients, and the liver injury is associated with the production of chemokines. Herein, we investigated the mechanism of how HAV infection induces the production of CXCR3 and CCR5 chemokines, such as CXCL10, CCL4 and CCL5. The production of CXCL10, CCL4 and CCL5 was markedly increased by HAV (HM-175/18f) infection in the culture of primary human hepatocytes and HepG2 cells. In particular, CXCL10 was produced in HAV-infected cells, not in neighboring uninfected cells. Moreover, these chemokines were significantly increased in the sera of acute hepatitis A patients. The production of IFN-λs was also robustly induced by HAV infection, and the blocking of secreted IFN-λs partially abrogated the production of CCL4 and CCL5 in HAV-infected cells. However, CXCL10 production was not decreased by the blocking of IFN-λs. Instead, CXCL10 production was reduced by silencing the expression of RIG-I-like receptor (RLR) signal molecules, such as mitochondrial antiviral signaling protein and interferon regulatory factor 3, in HAV-infected cells. In conclusion, HAV infection strongly induces the production of helper 1 T cell-associated chemokines, particularly CXCL10 via RLR signaling, even without secreted IFNs.

## Introduction

Hepatitis A virus (HAV), which belongs to the family *Picornaviridae*, is transmitted via fecal to oral routes and is endemic in developing countries^1, 2^. Primary HAV infection tends to be asymptomatic in children but often causes acute hepatitis A (AHA) accompanied with severe liver injury in adults^3^. In AHA patients, the virus is eliminated after extensive immune-mediated liver injury^4^, and a lifelong immunity is established. Inactivated virus-based vaccines are now available in developed countries, and vaccination results in a dramatic decline of the incidence of AHA in these countries^2, 5^.

After the picornaviral infection of host cells, cytosolic viral dsRNA intermediates are recognized by melanoma differentiation-associated protein 5 (MDA-5), which belongs to retinoic acid-inducible gene-I (RIG-I)-like receptors (RLRs), and endosomal dsRNA intermediates are recognized by Toll-like receptor 3 (TLR3)^6–8^. Intracellular signals from RLRs are transmitted via an adaptor protein called mitochondrial antiviral signaling protein (MAVS), thus leading to the interferon regulatory factor 3 (IRF3)- and nuclear factor kappa B (NF-κB)-dependent production of type I and III interferons (IFNs) and proinflammatory cytokines^6, 7, 9^.

Despite this mechanism of IFN induction, HAV is known to minimally stimulate IFN response in the infected liver^3^. In chimpanzee studies, the amount of viral RNA is substantially higher in the HAV-infected liver compared to the hepatitis C virus (HCV)-infected liver. However, a type I IFN response is barely detected in the HAV-infected liver, whereas it is robustly evoked in the HCV-infected liver^10^. This may be because HAV has several mechanisms that strongly impair the induction of IFNs in the infected cells^4, 11–14^. First, 3ABC, which is an intermediate product of HAV polyprotein processing, targets MAVS for proteolysis^12^. HAV also cleaves TRIF via another precursor, 3CD, thus resulting in interference with TLR3 signaling^11^, and HAV 3C protease cleaves the NF-κB essential modulator (NEMO), which is an upstream molecule in the NF-κB pathway^14^. Collectively, HAV antagonizes the innate immune response triggered by its dsRNA intermediates, which leads to the abrogation of type I IFN production in infected cells. Although plasmacytoid dendritic cells (pDCs) can produce type I IFNs by sensing enveloped HAV particles, they disappear from the liver two weeks after peak viremia^15^, which indicates that a pDC-dependent type I IFN response is transient during HAV infection.

In AHA, severe liver injury is associated with the infiltration of immune cells to the liver^4, 16–18^. Immune cells are recruited to peripheral inflammatory tissues through the action of various chemokines^19^. Notably, the expression of CXCL10, a representative CXCR3 chemokine, is elevated in the early stage of HAV infection in chimpanzees^10^. Moreover, various chemokines are significantly increased in the sera of AHA patients, including CXCL10, CCL4 and CCL5^20, 21^, which recruit CXCR3- or CCR5-expressing cytotoxic CD8^+^ and helper 1 CD4^+^ T cells. In virus-infected cells, the production of these chemokines is usually stimulated by type I IFNs^22–25^. This led us to investigate the mechanism of how chemokines are produced despite a minimal type I IFN response in HAV-infected cells. We found that HAV infection robustly induces the production of CXCR3 and CCR5 chemokines, such as CXCL10, CCL4 and CCL5, and demonstrated that in HAV-infected cells, CXCL10 is produced in a MAVS- and IRF3-dependent but IFN-independent manner.

## Results

### CXCL10, CCL4, and CCL5 are robustly produced from HAV-infected cells

**Figure 1:**
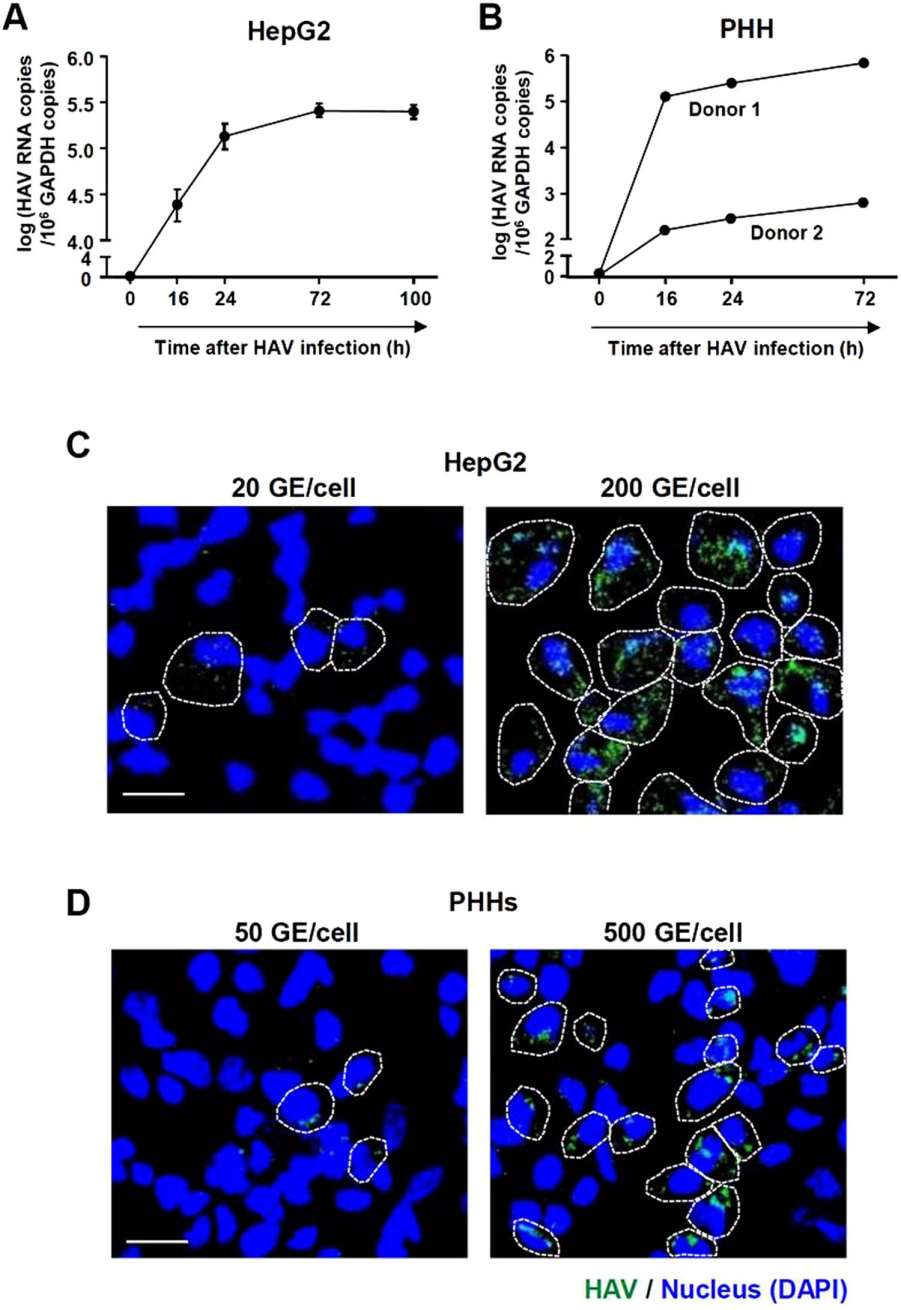
Infection of HM-175/18 f HAV in HepG2 cells and PHHs. (**A**,**B**) HepG2 cells were infected with HM-175/18 f HAV at 200 GE/cell (**A**), and PHHs from two different donors were infected at 500 GE/cell (**B**). Cell pellets were harvested and real-time qPCR was performed to examine intracellular HAV RNA copies. Bar graphs represent the means ± s.e.m. Each experiment was performed in triplicates. (**C**,**D**) HepG2 cells were infected with HM-175/18f HAV at 20 GE/cell or 200 GE/cell (**C**), and PHHs were infected at 50 GE/cell or 500 GE/cell (**D**). After 48 hours, immunofluorescence staining was performed to identify HAV-infected cells. Nucleus was stained with DAPI. Scale bar represents 20 μm. HAV antigen-positive cells are demarcated by dashed lines.

**Figure 2:**
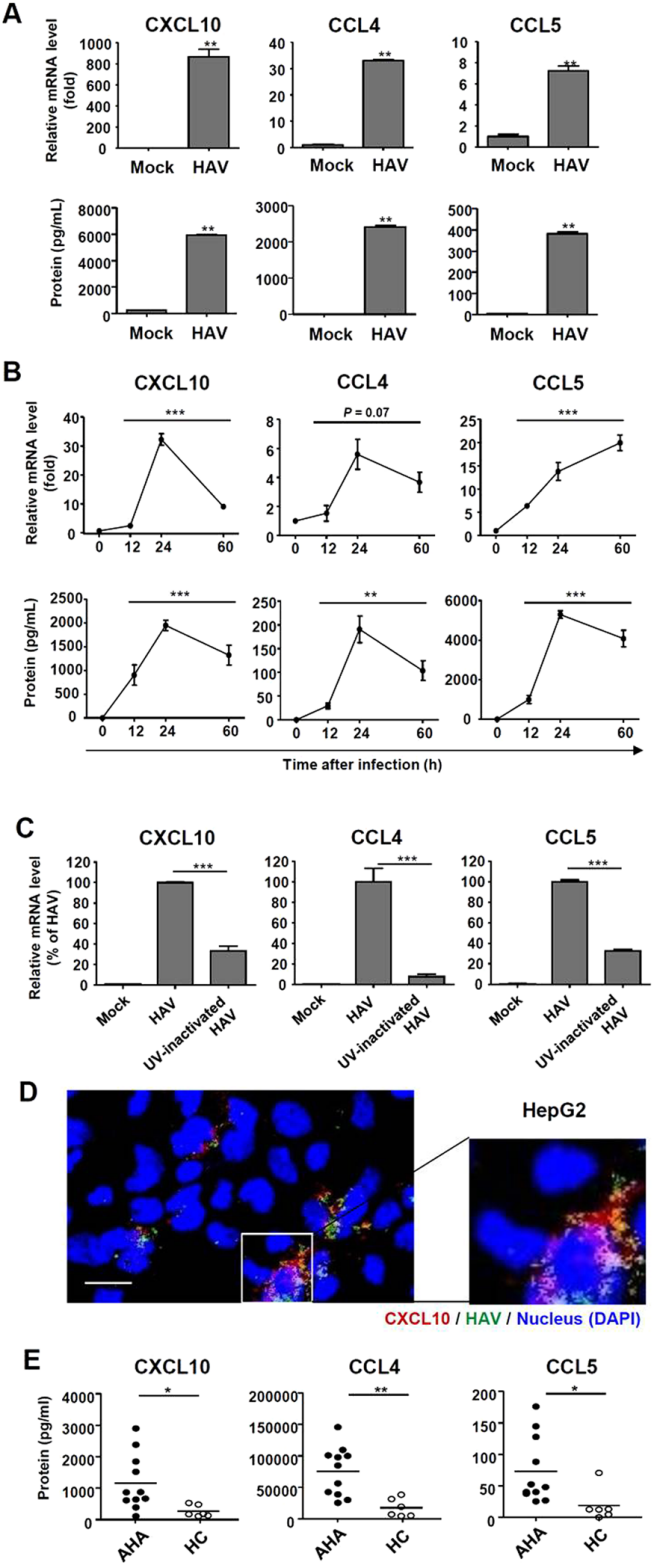
Production of CXCL10, CCL4, and CCL5 in HAV-infected cells. (**A**) PHHs were infected with HAV at 500 GE/cell. After 24 hours, cell pellets and culture supernatants were harvested. Real-time qPCR (upper row) and ELISA (lower row) were performed to examine the production of CXCL10, CCL4, and CCL5. Bar graphs represent the means ± s.e.m. (n = 3). Unpaired t-tests were performed. **P < 0.01 compared to mock infection. (**B**) HepG2 cells were infected with HAV at 200 GE/cell. Cell pellets and culture supernatants were harvested after HAV infection. Real-time qPCR (upper row) and ELISA (lower row) were performed to examine the production of CXCL10, CCL4, and CCL5. Means ± s.e.m. are shown (n = 3). Repeated measures ANOVA tests were performed. **P < 0.01, ***P < 0.001. (**C**) HepG2 cells were infected with mock, HAV, or UV-inactivated HAV at 200 GE/cells. After 30 hours, cell pellets were harvested. Real-time qPCR was performed to examine the induction of CXCL10, CCL4, and CCL5. Bar graphs represent the means ± s.e.m. (n = 3). Unpaired t-tests were performed. ***P < 0.001 compared to HAV infection. (**D**) HepG2 cells were infected with HM-175/18 f HAV at 50 GE/cell. After 48 hours, immunofluorescence staining was performed to examine the expression of CXCL10 and HAV antigen. Nucleus was stained with DAPI. Data from three independent experiments are presented. Scale bar represents 10 μm. (**E**) Sera from acute hepatitis A (AHA) patients (n = 11) and healthy controls (HC) (n = 6) were analyzed for CXCL10, CCL4, and CCL5 protein. Bar graphs represent the means ± s.d. Unpaired t-tests were performed. *P < 0.05, **P < 0.01 compared to HC.

In the present study, we used the HM-175/18f strain of HAV. HepG2 cells and PHHs from two different donors were inoculated with high titer HAV (200 GE/cell for HepG2 and 500 GE/cell for PHHs) and robust replication of HAV was observed in these cells (Fig. 1A and B). In immunofluorescence staining, almost all of the HepG2 cells (Fig. 1C) and around 50% of the PHHs (Fig. 1D) expressed HAV antigens after the infection at 200 GE/cell and 500 GE/cell, respectively. Of note, the signal of HAV antigens in each infected cell was much less in PHHs than in HepG2 cells (Fig. 1C and D). Next, we determined whether HAV infection induces the production of chemokines for CXCR3 and CCR5, which are chemokine receptors expressed by cytotoxic CD8^+^ T cells and helper 1 CD4^+^ T cells. In HAV-infected PHHs, the robust production of CXCL10, CCL4 and CCL5 was observed at the mRNA and protein levels (Fig. 2A), and these data were confirmed in HAV-infected HepG2 cells (Fig. 2B). UV-inactivated, replication-defective HAV induced chemokine expression in significantly less extent compared with replication-competent virus (Fig. 2C). Immunofluorescence co-staining revealed that CXCL10 was expressed in HAV-infected cells, but not in uninfected neighboring cells (Fig. 2D and Supplementary Fig. 1). We also studied CXCL10, CCL4 and CCL5 in the serum samples of AHA patients. We found that these chemokines were significantly increased in the sera from AHA patients compared to those from healthy controls (Fig. 2E). Collectively, these data show that HAV infection causes the robust production of CXCL10, CCL4, and CCL5.

### IFN-λs are produced from HAV-infected cells

**Figure 3:**
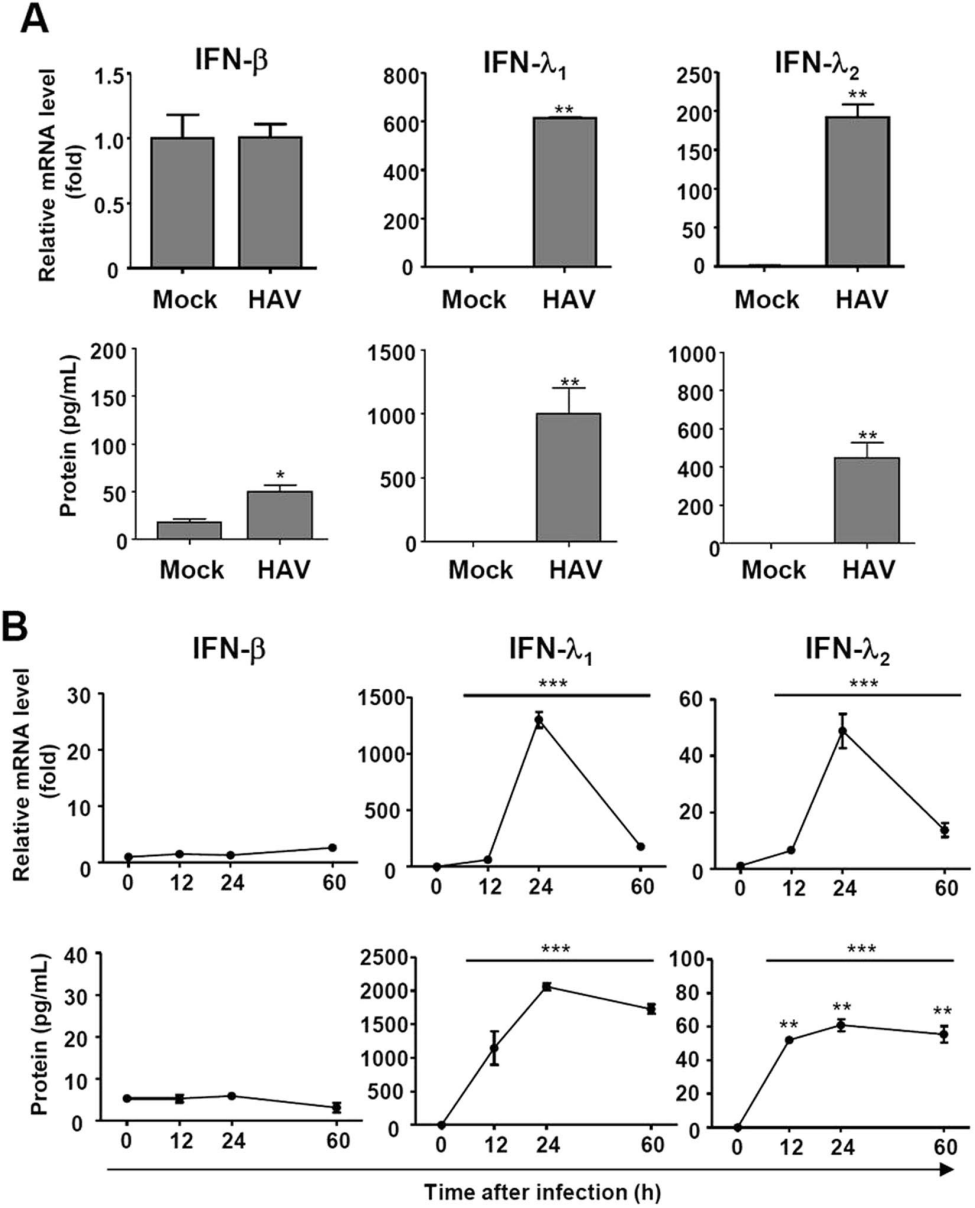
Production of IFN-λs in HAV-infected cells. (**A**) PHHs were infected with HAV at 500 GE/cell. After 24 hours, cell pellets and culture supernatants were harvested. Real-time qPCR (upper row) and ELISA (lower row) were performed to examine the production of IFN-β and -λs. Bar graphs represent the means ± s.e.m. (n = 3). Unpaired t-tests were performed. *P *<* 0.05, **P < 0.01 compared to mock infection. (**B**) HepG2 cells were infected with HAV at 200 GE/cell. Cell pellets and culture supernatants were harvested after HAV infection. Real-time qPCR (upper row) and ELISA (lower row) were performed to examine the production of IFN-β and -λs. Means ± s.e.m. are shown (n = 3). Repeated measures ANOVA tests were performed. ***P < 0.001.

Next, we examined the production of IFN-β and IFN-λs in HAV-infected cells because the production of CXCR3 chemokines and CCR5 chemokines can be induced by IFNs^23, 25–27^. After HAV infection, the production of IFN-λ_1_ and -λ_2_ was strongly induced at the mRNA and protein levels in PHHs (Fig. 3A). However, IFN-β was minimally produced after HAV infection (Fig. 3A). We also studied IFN production in HepG2 cells and found that HAV-infected HepG2 cells produced IFN-λ_1_ and -λ_2_ but not IFN-β in the early stage of infection (Fig. 3B). Collectively, these data demonstrate that HAV-infected cells robustly produce IFN-λs rather than IFN-β.

### Neutralization of IFN-λs partially abrogates CCL4 and CCL5 production but not CXCL10 production in HAV-infected cells

**Figure 4:**
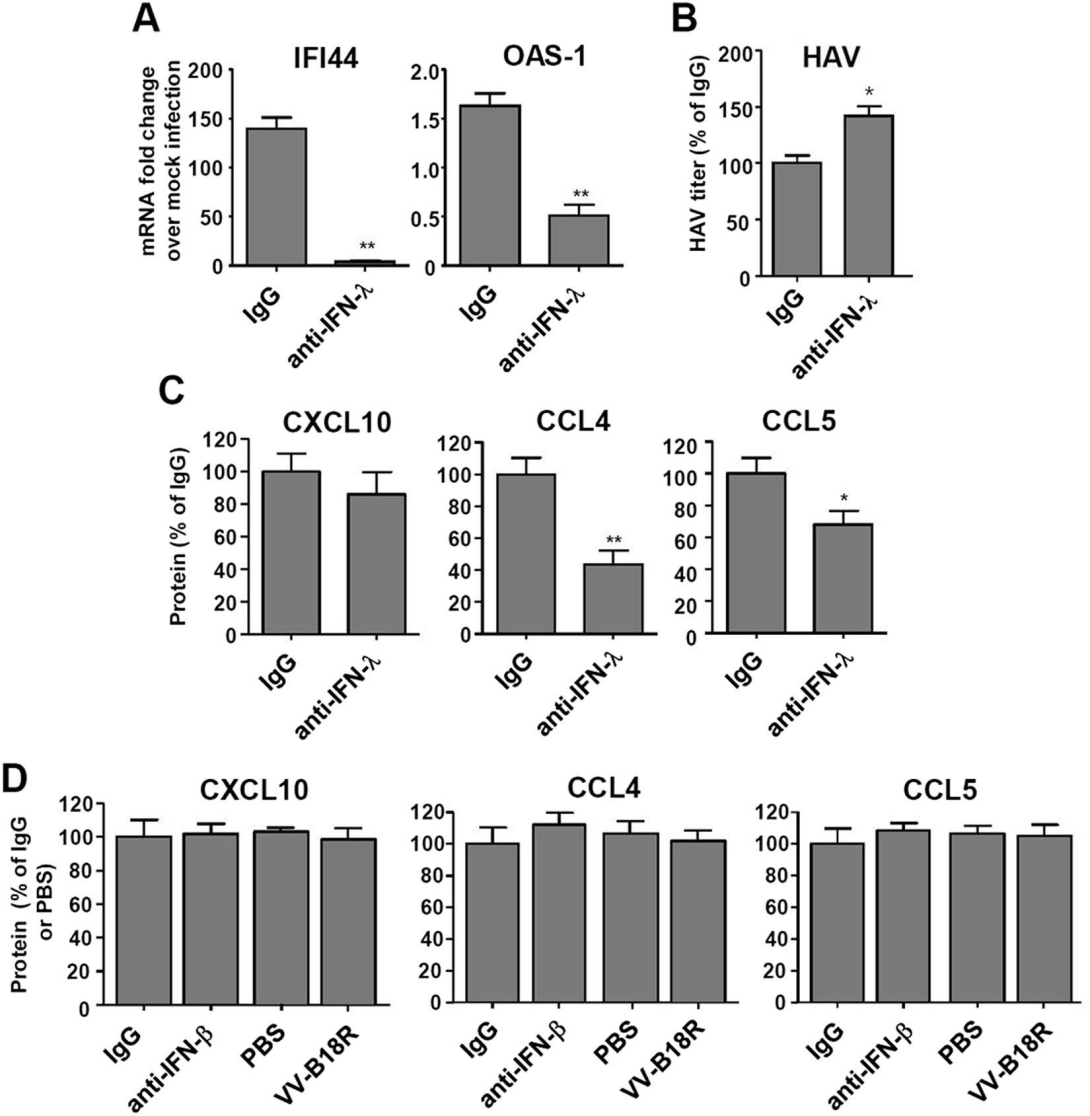
Effects of neutralizing IFN-λ on the production of chemokines in HAV-infected cells. (**A**–**D**) Anti-IFN-λ, anti-IFN-β, or VV-B18R was added to the HepG2 cell culture 30 minutes prior to HAV infection (200 GE/cell), and maintained until harvesting. After 48-hour culture, real-time qPCR was performed to examine the effect of anti-IFN-λ on IFI44 and OAS-1 induction (**A**). After 48-hour culture, real-time qPCR was performed to examine intracellular HAV RNA copies (**B**). After 24-hour culture, culture supernatants were harvested and ELISA was performed to examine the production of CXCL10, CCL4, and CCL5 (**C**,**D**). Bar graphs represent the means ± s.e.m. (n = 3). Unpaired t-tests were performed. *P < 0.05, **P < 0.01 compared to PBS or IgG.

IFN-λs were potently produced from HAV-infected cells. Thus, we investigated whether IFN-λs are responsible for the production of chemokines. We blocked the effect of IFN-λs by using neutralizing anti-IFN-λ antibody at a high dose, at a concentration sufficient to neutralize all three IFN-λs, IFN-λ_1~3_^28^. The neutralization of IFN-λs abrogated the induction of interferon-stimulated genes (ISGs) such as IFI44 and OAS-1 in HAV-infected HepG2 cells (Fig. 4A). Accordingly, intracellular HAV RNA titer was slightly increased by neutralization of IFN-λs (Fig. 4B). However, the production of CXCL10 was not decreased by the neutralization of IFN-λs (Fig. 4C). Differently from CXCL10, production of CCL4 and CCL5 was partially abrogated by the neutralization of IFN-λs (Fig. 4C). As expected from the aforementioned result (IFN-β was not produced from HAV-infected HepG2 cells (Fig. 3B)), the production of chemokines was not decreased by neutralizing anti-IFN-β antibody or the vaccinia virus–encoded B18 receptor protein (VV-B18R), which competes with the IFN-α/βR for IFN binding^29^ (Fig. 4D). The neutralizing activity of anti-IFN-β antibody was confirmed by spiking experiments using recombinant IFN-β (Supplementary Fig. 2). These results indicate that in HAV-infected cells, CCL4 and CCL5 are induced, at least in part, by IFN-λs, whereas CXCL10 is induced independently of IFN-λs.

### Silencing of MAVS or IRF3 expression abrogates the production of chemokines in HAV-infected cells

**Figure 5:**
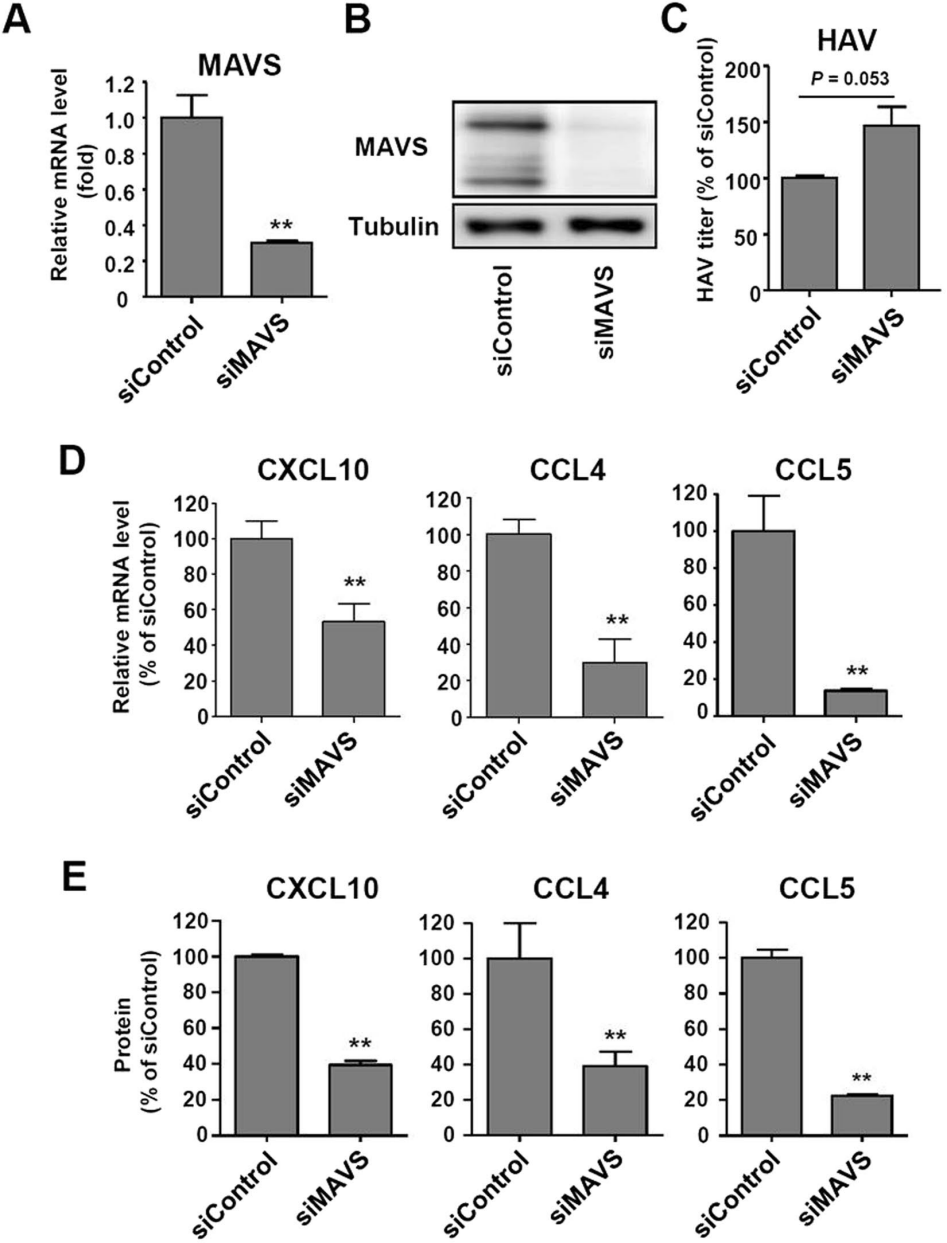
MAVS-dependent production of CXCL10, CCL4, and CCL5 in HAV-infected cells. (**A**,**B**) HepG2 cells were transfected with siRNA targeting scrambled sequences (siControl) or MAVS (siMAVS). Efficient knock-down by siMAVS was confirmed via real-time qPCR (**A**) and immunoblotting (**B**). Bar graphs represent the means ± s.e.m. (n = 3). Unpaired t-tests were performed. **P < 0.01 compared to siControl. (**C**–**E**) HepG2 cells were transfected with siControl or siMAVS. After 72 hours, the cells were infected with HAV at 200 GE/cell. Cell pellets and culture supernatants were harvested 24 hours after infection. Intracellular HAV RNA titer was examined by real-time qPCR (**C**). Real-time qPCR (**D**) and ELISA (**E**) were performed to examine the production of CXCL10, CCL4, and CCL5. Bar graphs represent the means ± s.e.m. (n = 3). Unpaired t-tests were performed. **P < 0.01 compared to siControl.

In HCV-infected cells, the CXCL10 promoter was recently shown to be activated by IRF3 independently of type I or III IFNs^30^. Therefore, we investigated whether CXCL10 is produced by a similar mechanism in HAV-infected cells. First, we tested if RIG-I or MDA-5 involved in the production of CXCL10 in HAV-infected cells. We found that the expression of CXCL10 was decreased by MDA-5 silencing (Supplementary Fig. 3), but not by RIG-I silencing (Supplementary Fig. 4). Our data are consistent with previous reports, which demonstrated that picornaviruses are recognized by MDA-5, not by RIG-I, leading to MAVS activation^7, 8^. Next, we knocked down the expression of MAVS, a downstream of RLRs and an upstream of IRF3^31, 32^, via siRNA transfection in HepG2 cells. The efficient silencing of MAVS expression was confirmed at both the mRNA and protein levels (Fig. 5A and B). In HAV infection experiments, intracellular HAV RNA titer tended to be increased by MAVS silencing although the difference was not statistically significant (Fig. 5C). Importantly, the production of CXCL10, CCL4 and CCL5 was significantly decreased by MAVS silencing at the both mRNA and protein levels; however, it was not completely abolished (Fig. 5D and E).

**Figure 6:**
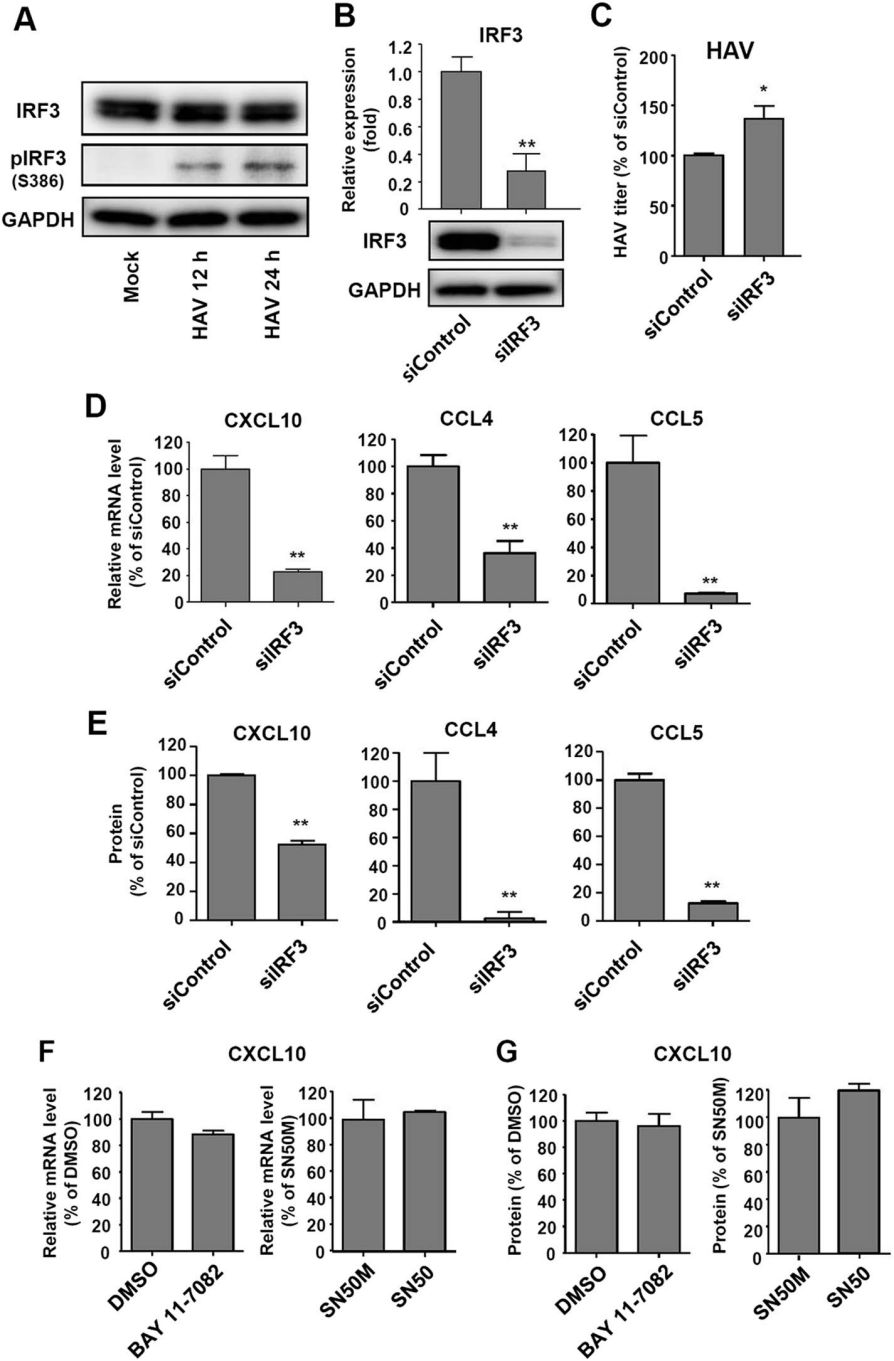
IRF3-dependent production of CCL4, CCL5, and CXCL10 in HAV-infected cells. (**A**) HepG2 cells were infected with HAV at 500 GE/cell. Cell pellets were harvested 12 and 24 hours after infection. IRF3 and phospho-IRF3 (S386) were detected via immunoblotting. Data are representative of two independent experiments. (**B**) HepG2 cells were transfected with siControl or siRNA targeting IRF3 (siIRF3). Efficient knock-down by siIRF3 was confirmed via real-time qPCR (upper) and immunoblotting (lower). Bar graphs represent the means ± s.e.m. (n = 3). Unpaired t-tests were performed. **P < 0.01 compared to siControl. (**C**–**E**) HepG2 cells were transfected with siControl or siIRF3. After 72 hours, cells were infected with HAV at 200 GE/cell. Cell pellets and culture supernatants were harvested 24 hours after infection. Intracellular HAV RNA titer was examined by real-time qPCR (**C**). Real-time qPCR (**D**) and ELISA (**E**) were performed to examine the production of CXCL10, CCL4, and CCL5. Bar graphs represent the means ± s.e.m. (n = 3). Unpaired t-tests were performed. *P < 0.05, **P < 0.01 compared to siControl. (**F**,**G**) HepG2 cells were treated with 2 μM BAY 11–7082 or DMSO, or 50 mg/L SN50M or SN50. After 24 hours, cells were infected with HAV at 200 GE/cell. Cell pellets and culture supernatants were harvested 24 hours after HAV infection. Real-time qPCR (**F**) and ELISA (**G**) were performed to examine the production of CXCL10. Bar graphs represent the means ± s.e.m. (n = 3). Unpaired t-tests were performed.

Next, we examined the role of IRF3 in the production of chemokines in HAV-infected cells. Phosphorylation at S386 in its C-terminal regulatory region is a major determinant of IRF3 activation^33–35^.We found that HAV infection stimulates IRF3 phosphorylation at S386 in HepG2 cells (Fig. 6A). IRF3 expression was efficiently silenced by siRNA at both the mRNA and protein levels (Fig. 6B). Intracellular HAV RNA titer was increased by IRF3 silencing (Fig. 6C). The production of CXCL10, CCL4 and CCL5 was significantly decreased in IRF3-silenced, HAV-infected HepG2 cells at both the mRNA and protein levels; however, the production of CXCL10 protein was not completely abrogated (Fig. 6D and E). We also tested if NF-κB pathway involved in the production of CXCL10 in HAV-infected cells using a chemical inhibitor (BAY 11–7082) and a peptide inhibitor (SN50). However, both of these inhibitors did not reduce HAV-induced production of CXCL10 (Fig. 6F and G). Taken together, we conclude that CXCL10 is produced in HAV-infected cells in a MAVS and IRF3-dependent but IFN-independent manner.

## Discussion

In the present study, we examined the production of CXCR3 and CCR5 chemokines and their expression mechanisms in HAV-infected cells. We demonstrated that CXCL10, CCL4 and CCL5 are robustly produced in HAV-infected cells. CCL4 and CCL5 are produced, at least in part, by secreted IFN-λs after HAV infection. However, CXCL10 is produced by a MAVS- and IRF3-dependent mechanism independently of IFNs.

A similar mechanism of CXCL10 production was recently reported in HCV infection. In HCV-infected Huh-7-TLR3 cells, CXCL10 production was independent of type I and III IFNs^36^. Instead, CXCL10 expression is directly regulated by IRF3 and NF-κB^30^. Notably, the CXCL10 promoter contains binding sites for IRF3 and NF-κB, and its transcriptional activity is directly activated by IRF3 and NF-κB^30^. Regarding the role of NF-κB, however, our current result with HAV-infected cells differs from the previous report with HCV-infected cells. Both of the NF-κB inhibitors (BAY 11–7082 and SN50) did not reduce HAV-induced production of CXCL10 (Fig. 6F and G), indicating that CXCL10 production is independent of NF-κB activation in HAV-infected cells unlike in HCV-infected cells. Our data is consistent with a recent report showing that the phospho-p65 component of NF-κB was not measurably increased in HAV-infected livers of mice lacking type I IFN receptor that support HAV replication and recapitulate the typical features of AHA in humans^37^.

HAV is known to evade the IFN response of the host. HAV disrupts the intracellular signals of RLRs by targeting MAVS for proteolysis through a precursor of its cysteine protease, 3ABC^12^. HAV also inhibits TLR3 signaling by cleaving TRIF via another precursor, 3CD^11^. However, our data indicate that HAV cannot completely suppress the innate response of hepatocytes. First, MAVS was not completely cleaved in HAV-infected HepG2 cells in the early stage of infection (data not shown). Incomplete MAVS cleavage might be due to the low level of viral protease proteins in the early stage of HAV infection. Second, the phosphorylation of IRF3 at S386 was observed in HAV-infected cells (Fig. 6A). Third, IFN-λs were robustly produced in PHHs after HAV infection (Fig. 3A). Whereas the production of IFN-λs after HCV infection was described in primary liver cell cultures^38^, PHHs^27, 39^, CD81- and mir-122-expressing HepG2 cells^40^, and Huh-7-TLR3 cells^28^, it should be noted that IFN-λ production in HAV-infected cells was firstly demonstrated in the present study. Due to this incomplete evasion from RLR signaling, CCL4 and CCL5 are produced by MAVS-, IRF3- and IFN-λ-dependent mechanisms in HAV-infected cells, and CXCL10 is produced by a MAVS- and IRF3-dependent but IFN-independent mechanism. Incomplete evasion from RLR signaling and the resultant production of chemokines have been similarly observed in HCV-infected cells and in HCV-infected chimpanzees^30, 36^.

Recently, it was demonstrated that mice lacking type I IFN receptor are permissive to HAV infection, recapitulating the typical features of AHA in humans^37^. The authors of this report suggested that the restricted host range of HAV results from an inability to hamper MAVS-mediated type I IFN production^37^. Their results are consistent with our current data in that HAV infection causes IRF3 phosphorylation and chemokine production independently of type I IFNs^37^. Their data are also consistent with our current data in that MAVS- or IRF3-knockout mice did not produce chemokines. However, our current study used materials originated from humans, e.g., human PHHs, human liver-derived cells and human patients’ sera. Moreover, we demonstrate that type III IFNs, rather than type I IFNs, are dominantly produced from human liver-derived cells after HAV infection^37^.

Acute HAV infection causes necroinflammatory liver injury, particularly in adult patients, and is characterized by the infiltration of numerous immune cells^4, 16–18^. The role of inflammatory chemokines in the recruitment of immune cells and liver injury has been thoroughly investigated in HCV infection^23, 26, 41^. Among various chemokines, CXCR3 chemokines and CCR5 chemokines have been extensively studied in viral hepatitis because they play a major role in T-cell recruitment to peripheral inflammatory sites^26, 42^. In particular, CXCR3 and CCR5 are involved in the recruitment of effector CD8^+^ T cells and helper 1 CD4^+^ T cells into the liver parenchyma and portal tracts, respectively, in HCV-infected livers^43–45^. Moreover, the expression levels of CXCR3 and CCR5 chemokines in the liver or the peripheral blood strongly correlate with the severity of hepatic inflammation in HCV infection^46–49^. Thus, CXCR3 and CCR5 chemokines may also play a critical role in hepatic inflammation in acute HAV infection. Notably, the expression of CXCL10 is upregulated in the early stage of HAV infection in chimpanzees^10^, and CXCL10, CCL4 and CCL5 are significantly increased in the sera of AHA patients (Fig. 2E)^21^.

In the present study, we showed the robust production of these chemokines in HAV-infected cells and elucidated the underlying mechanisms. This study provides useful information for regulating the expression of inflammatory chemokines, which are related to liver injury in HAV infection.

## Materials and Methods

### Cells and reagents

Huh-7.5 cells (Apath, Brooklyn, NY) and HepG2 cells (ATCC, Manassas, VA) were maintained at 37 °C with 5% CO_2_ in Dulbecco’s Modified Eagle Medium supplemented with 10% fetal bovine serum (WELGENE, Daegu, Korea), 4.5 g/L glucose, L-glutamine, and 1% penicillin/streptomycin (Invitrogen, Carlsbad, CA). Frozen vials of primary human hepatocytes (PHHs) were purchased from Invitrogen. After thawing, the PHHs were centrifuged at 150 × g for 5 minutes and incubated in 24-well plates overnight. The PHHs were maintained in Williams E Medium containing cell maintenance supplement reagents (Invitrogen).

Small interfering RNAs (siRNAs) against MAVS, IRF3, MDA-5, and RIG-I were obtained from Santa Cruz Biotechnology (Santa Cruz, CA), and siRNA transfection was performed using Lipofectamine RNAi MAX (Invitrogen). Neutralizing antibody against IFN-λ was purchased from R&D Systems (Minneapolis, MN) and used at 20 μg/mL, the concentration at which it is sufficient to neutralize IFN-λ_1_, -λ_2_ and -λ_3_^28^. Neutralizing anti-IFN-β antibody and VV-B18R protein were purchased from PBL Assay Science (Piscataway, NJ) and eBioscience (San Diego, CA), respectively. An IFN-β enzyme-linked immunosorbent assay (ELISA) kit was purchased from Fujirebio (Tokyo, Japan), and IL-28A (IFN-λ_2_) and IL-29 (IFN-λ_1_) ELISA kits were purchased from RayBiotech (Norcross, GA). BAY 11–7082, an NF-κB chemical inhibitor was purchased from Calbiochem (San Diego, CA), and SN50, an NF-κB peptide inhibitor and its control peptide SN50M were purchased from Biomol Research Laboratories (Plymouth Meeting, PA).

### HAV propagation and infection

A rapidly replicating, cell culture-adapted, cytopathic variant of HM-175 (HM-175/18f) HAV^50, 51^ was obtained from American Type Culture Collection (ATCC, VR-1402). For virus propagation, Huh-7.5 cells were infected with HM-175/18f virus at 5 genome equivalents (GE)/cell and maintained for more than 14 days. Viral stock was prepared after rendering infected cells to repeated free-thaw cycles. Virus-containing medium was concentrated with WELPROT^TM^ virus concentration reagent (WELGENE). Chemokine induction was studied with PHHs and HepG2 cells. HepG2 cells are known to support HAV infection and replication^52^. HepG2 cells and PHHs were infected with HM-175/18f virus at 200 GE/cell and 500 GE/cell, respectively.

### Serum samples of AHA patients

Eleven hospitalized patients diagnosed with AHA were recruited for the study. The eligibility criteria of this study included seropositivity for anti-HAV IgM and IgG antibodies, serum alanine aminotransferase (ALT) levels higher than three-fold the upper normal limit, and clinical manifestations consistent with acute hepatitis. This study was performed according to the ethical guidelines of the Declaration of Helsinki and was approved by the Institutional Review Board of KAIST. Written informed consents were obtained from all of the participants.

### Cytometric bead array

The concentrations of chemokines (CXCL10, CCL4, and CCL5) in culture supernatants and patients’ sera were measured via cytometric bead array (CBA). Briefly, 50 μL of mixed capture beads and 50 μL of each sample were incubated for 1 hour at room temperature, and 50 μL of mixed phycoerythrin (PE) detection reagents was added to the bead-sample mixture and incubated for 2 hours at room temperature. The fluorescence of the beads was analyzed using an LSRII Flow Cytometer (BD Biosciences, San Jose, CA), and the data were analyzed using FlowJo software (Tree Star, San Carlos, CA).

### Immunoblotting

Immunoblotting was performed as previously described^53^. Briefly, the cell lysate was prepared using RIPA buffer, and 10 μg of the cell lysate was loaded onto SDS-PAGE gels and analyzed using immunoblots. The antibodies used in immunoblotting are as follows: mouse monoclonal anti-MAVS (Santa Cruz Biotechnology), rabbit monoclonal anti-IRF3 (clone EPR2418Y, Abcam, Cambridge, MA), rabbit monoclonal anti-phospho IRF3 (S386) (clone EPR2346, Abcam), rabbit polyclonal anti-GAPDH (Santa Cruz Biotechnology), and mouse monoclonal anti-tubulin (Sigma-Aldrich, St. Louis, MO).

### Confocal microscopy

Slide preparation and microscopic procedures were conducted as previously described^53^. Mouse monoclonal anti-HAV antibody (clone 7E7, Mediagnost GmbH, Reutlingen, Germany) and rabbit polyclonal anti-CXCL10 antibody (Abcam) were used as primary antibodies. Alexa Fluor^®^ 488-conjugated goat anti-mouse IgG and Alexa Fluor^®^ 594-conjugated goat anti-rabbit IgG (Invitrogen) were used as secondary antibodies. Nuclear staining was performed using Hoechst 33342 dye (Sigma-Aldrich).

### RNA extraction, cDNA synthesis, and real-time quantitative PCR

Total RNA isolation, cDNA synthesis, and TaqMan real-time quantitative PCR were performed as previously described^53^. In brief, total RNA was isolated with the RNeasy Mini kit (Qiagen, Valencia, CA), and cDNA was synthesized using the High Capacity cDNA Synthesis Kit (Applied Biosystems, Foster City, CA). TaqMan Gene Expression Assays (Applied Biosystems) were used to determine the mRNA levels of target genes. The results were standardized to the mRNA level of an endogenous control, β-actin. Sequences of primers for HAV RNA titration were adopted from a previous report^10^.

### Statistical analyses

Data from experiments using cell lines are presented as the means ± standard error of the means (s.e.m.). Unpaired t-tests or repeated-measures ANOVA were used to assess for statistical differences. All of the statistical analyses were conducted using GraphPad Prism version 5.01 (GraphPad Software, San Diego, CA). A *P* value less than 0.05 was considered statistically significant.

## Electronic supplementary material

Supplementary Information

## References

[1] Chung SJ (2014). Changes in the seroprevalence of IgG anti-hepatitis A virus between 2001 and 2013: experience at a single center in Korea. Clin Mol Hepatol.

[2] Collier MG, Tong X, Xu F (2015). Hepatitis A hospitalizations in the United States, 2002–2011. Hepatology.

[3] Shin EC, Sung PS, Park SH (2016). Immune responses and immunopathology in acute and chronic viral hepatitis. Nat Rev Immunol.

[4] Walker CM, Feng Z, Lemon SM (2015). Reassessing immune control of hepatitis A virus. Curr Opin Virol.

[5] Martin A, Lemon SM (2006). Hepatitis A virus: from discovery to vaccines. Hepatology.

[6] Debing Y, Neyts J, Thibaut HJ (2014). Molecular biology and inhibitors of hepatitis A virus. Med Res Rev.

[7] Feng Q, Langereis MA, van Kuppeveld FJ (2014). Induction and suppression of innate antiviral responses by picornaviruses. Cytokine Growth Factor Rev.

[8] Kato H (2006). Differential roles of MDA5 and RIG-I helicases in the recognition of RNA viruses. Nature.

[9] Park SH, Rehermann B (2014). Immune responses to HCV and other hepatitis viruses. Immunity.

[10] Lanford RE (2011). Acute hepatitis A virus infection is associated with a limited type I interferon response and persistence of intrahepatic viral RNA. Proc Natl Acad Sci U S A.

[11] Qu L (2011). Disruption of TLR3 signaling due to cleavage of TRIF by the hepatitis A virus protease-polymerase processing intermediate, 3CD. PLoS Pathog.

[12] Yang Y (2007). Disruption of innate immunity due to mitochondrial targeting of a picornaviral protease precursor. Proc Natl Acad Sci USA.

[13] Fensterl V (2005). Hepatitis A virus suppresses RIG-I-mediated IRF-3 activation to block induction of beta interferon. J Virol.

[14] Wang D (2014). Hepatitis A virus 3C protease cleaves NEMO to impair induction of beta interferon. J Virol.

[15] Feng Z (2015). Human pDCs preferentially sense enveloped hepatitis A virions. J Clin Invest.

[16] Siegl G, Weitz M (1993). Pathogenesis of hepatitis A: persistent viral infection as basis of an acute disease?. Microb Pathog.

[17] Koff, R. S. Hepatitis A. *Lancet* **351**, 1643–1649, doi:10.1016/S0140-6736(98)01304-X (1998). 10.1016/S0140-6736(98)01304-X 9620732

[18] Choi YS (2015). Liver injury in acute hepatitis A is associated with decreased frequency of regulatory T cells caused by Fas-mediated apoptosis. Gut.

[19] Moreau R (2016). Acute-on-chronic liver failure: a new syndrome in cirrhosis. Clin Mol Hepatol.

[20] Duffy D (2014). The ABCs of viral hepatitis that define biomarker signatures of acute viral hepatitis. Hepatology.

[21] Shin SY (2016). Comparative Analysis of Liver Injury-Associated Cytokines in Acute Hepatitis A and B. Yonsei Med J.

[22] Kelly-Scumpia KM (2010). Type I interferon signaling in hematopoietic cells is required for survival in mouse polymicrobial sepsis by regulating CXCL10. J Exp Med.

[23] Shin, E. C. *et al*. Delayed induction, not impaired recruitment, of specific CD8(+) T cells causes the late onset of acute hepatitis C. *Gastroenterology* **141**, 686–695, 695 e681, 10.1053/j.gastro.2011.05.006 (2011). 10.1053/j.gastro.2011.05.006 PMC3359650 21699897

[24] Weighardt H (2006). Type I IFN modulates host defense and late hyperinflammation in septic peritonitis. J Immunol.

[25] Trinchieri G (2010). Type I interferon: friend or foe?. J Exp Med.

[26] Kang W, Shin EC (2011). Clinical implications of chemokines in acute and chronic hepatitis C virus infection. Yonsei Med J.

[27] Park H (2012). IL-29 is the dominant type III interferon produced by hepatocytes during acute hepatitis C virus infection. Hepatology.

[28] Sung PS (2015). Roles of unphosphorylated ISGF3 in HCV infection and interferon responsiveness. Proc Natl Acad Sci USA.

[29] Symons JA, Alcami A, Smith GL (1995). Vaccinia virus encodes a soluble type I interferon receptor of novel structure and broad species specificity. Cell.

[30] Brownell J (2014). Direct, interferon-independent activation of the CXCL10 promoter by NF-kappaB and interferon regulatory factor 3 during hepatitis C virus infection. J Virol.

[31] Kawai T (2005). IPS-1, an adaptor triggering RIG-I- and Mda5-mediated type I interferon induction. Nat Immunol.

[32] Kato H (2008). Length-dependent recognition of double-stranded ribonucleic acids by retinoic acid-inducible gene-I and melanoma differentiation-associated gene 5. J Exp Med.

[33] Panne D, McWhirter SM, Maniatis T, Harrison SC (2007). Interferon regulatory factor 3 is regulated by a dual phosphorylation-dependent switch. J Biol Chem.

[34] Chen W (2008). Contribution of Ser386 and Ser396 to activation of interferon regulatory factor 3. J Mol Biol.

[35] Mori M (2004). Identification of Ser-386 of interferon regulatory factor 3 as critical target for inducible phosphorylation that determines activation. J Biol Chem.

[36] Brownell J (2013). Independent, parallel pathways to CXCL10 induction in HCV-infected hepatocytes. J Hepatol.

[37] Hirai-Yuki A (2016). MAVS-dependent host species range and pathogenicity of human hepatitis A virus. Science.

[38] Marukian S (2011). Hepatitis C virus induces interferon-lambda and interferon-stimulated genes in primary liver cultures. Hepatology.

[39] Thomas E (2012). HCV infection induces a unique hepatic innate immune response associated with robust production of type III interferons. Gastroenterology.

[40] Israelow B, Narbus CM, Sourisseau M, Evans MJ (2014). HepG2 cells mount an effective antiviral interferon-lambda based innate immune response to hepatitis C virus infection. Hepatology.

[41] Zeremski M (2011). Induction of CXCR3- and CCR5-associated chemokines during acute hepatitis C virus infection. J Hepatol.

[42] Zeremski M, Petrovic LM, Talal AH (2007). The role of chemokines as inflammatory mediators in chronic hepatitis C virus infection. J Viral Hepat.

[43] Ajuebor MN, Hogaboam CM, Le T, Proudfoot AE, Swain MG (2004). CCL3/MIP-1alpha is pro-inflammatory in murine T cell-mediated hepatitis by recruiting CCR1-expressing CD4(+) T cells to the liver. Eur J Immunol.

[44] Curbishley SM, Eksteen B, Gladue RP, Lalor P, Adams DH (2005). CXCR 3 activation promotes lymphocyte transendothelial migration across human hepatic endothelium under fluid flow. Am J Pathol.

[45] Murai M (1999). Active participation of CCR5(+)CD8(+) T lymphocytes in the pathogenesis of liver injury in graft-versus-host disease. J Clin Invest.

[46] Apolinario A (2002). Increased expression of T cell chemokines and their receptors in chronic hepatitis C: relationship with the histological activity of liver disease. Am J Gastroenterol.

[47] Harvey CE (2003). Expression of the chemokine IP-10 (CXCL10) by hepatocytes in chronic hepatitis C virus infection correlates with histological severity and lobular inflammation. J Leukoc Biol.

[48] Helbig KJ (2004). Expression of the CXCR3 ligand I-TAC by hepatocytes in chronic hepatitis C and its correlation with hepatic inflammation. Hepatology.

[49] Zeremski M (2008). Intrahepatic levels of CXCR3-associated chemokines correlate with liver inflammation and fibrosis in chronic hepatitis C. Hepatology.

[50] Zhang H (1995). An infectious cDNA clone of a cytopathic hepatitis A virus: genomic regions associated with rapid replication and cytopathic effect. Virology.

[51] Feng Z (2013). A pathogenic picornavirus acquires an envelope by hijacking cellular membranes. Nature.

[52] Seggewiss N, Paulmann D, Dotzauer A (2015). Lysosomes serve as a platform for hepatitis A virus particle maturation and nonlytic release. Arch Virol.

[53] Sung PS (2014). Hepatitis C virus entry is impaired by claudin-1 downregulation in diacylglycerol acyltransferase-1-deficient cells. J Virol.

