# Supplementary material for: CXCL10 is produced in hepatitis A virus-infected cells in an IRF3-dependent but IFN-independent manner
Source: Sci Rep. 2017 Jul 25;7:6387. doi: 10.1038/s41598-017-06784-x (PMC5527116; doi:10.1038/s41598-017-06784-x)
Supplement: Supplementary file 1 — Supplementary Information [file 41598_2017_6784_MOESM1_ESM.pdf]

SREP-16-38063 Supplementary Information

CXCL10 is produced in hepatitis A virus-infected cells in an IRF3-dependent but IFN-independent manner

Pil Soo Sung[1, 2], Seon-Hui Hong[3], Jeewon Lee[3], Su-Hyung Park[3, 4], Seung Kew Yoon[2], Woo Jin Chung[5], Eui-Cheol Shin[1, 3 *]

[1]Laboratory of Immunology and Infectious Diseases, Graduate School of Medical Science and Engineering, KAIST, Daejeon, Republic of Korea

[2]Division of Hepatology, Department of Internal Medicine, Seoul St. Mary's Hospital, The Catholic University of Korea, Seoul, Republic of Korea

[3]Biomedical Science and Engineering Interdisciplinary Program, KAIST, Daejeon, Republic of Korea

[4]Laboratory of Translational Immunology and Vaccinology, Graduate School of Medical Science and Engineering, KAIST, Daejeon, Republic of Korea

[5]Department of Internal Medicine, Keimyung University School of Medicine, Daegu, Republic of Korea

Supplementary Figure 1

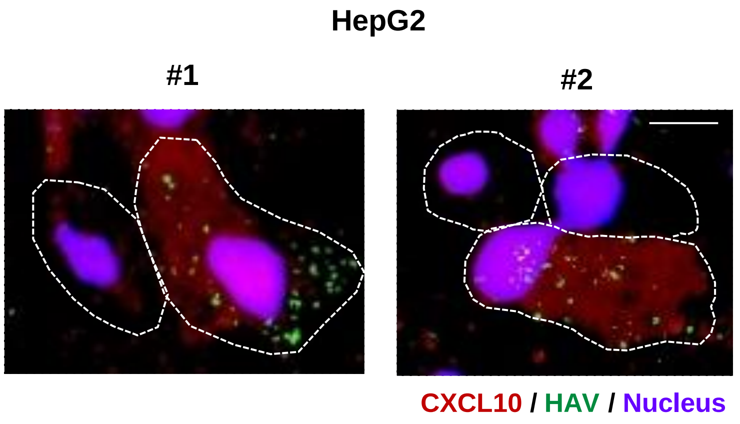

Supplementary Figure 1. HAV-infected HepG2 cells produce CXCL10.
HepG2 cells were infected with HM-175/18f HAV at 50 GE/cell. After 48 hours, immunofluorescence staining was performed to examine the expression of CXCL10 and HAV antigen. Nucleus was stained with DAPI. Data from two independent experiments are presented. Scale bar represents 10 μm. Cell margin is demarcated by dashed lines.

Supplementary Figure 2

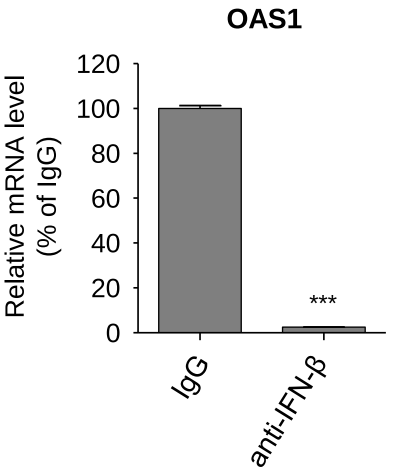

Supplementary Figure 2. IFN-β activity is neutralized by anti-IFN-β antibody. Huh-7 cells were pre-treated with anti-IFN-β antibody 30 minutes before treatment of 10 ng/mL recombinant IFN-β. After 48 hours, cells were harvested, and OAS1 gene expression was analysed by real-time qPCR. Data are presented as means ± s.e.m. (n = 3). ****P* ≤ 0.001 (Student's t-test).

Supplementary Figure 3

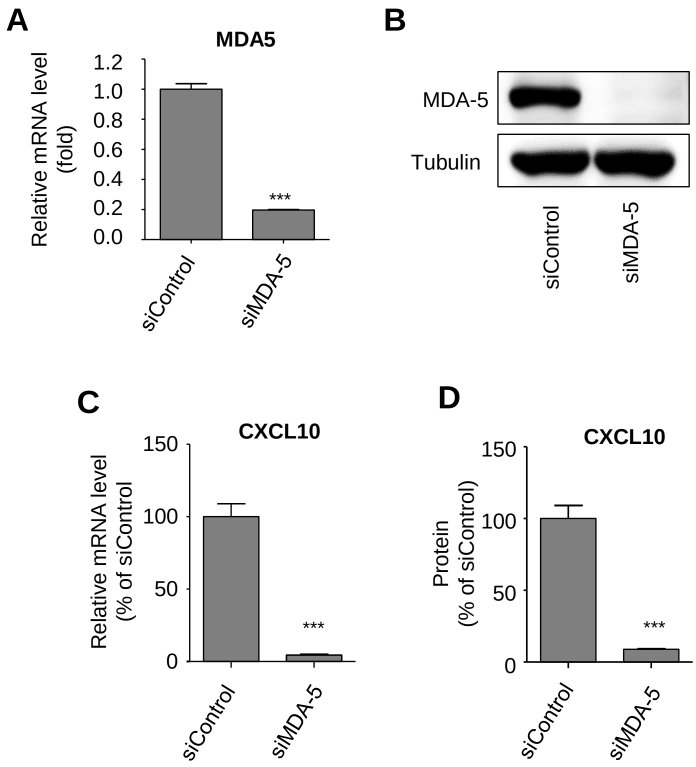

Supplementary Figure 3. MDA-5-dependent production of CXCL10 in HAV-infected cells.
(A-B) HepG2 cells were transfected with siRNA targeting scrambled sequences (siControl) or MDA-5 (siMDA-5). Efficient knock-down by siMDA-5 was confirmed via real-time qPCR (A) and immunoblotting (B). Bar graphs represent the means ± s.e.m. (n = 3). Unpaired t-tests were performed. ****P* < 0.001 compared to siControl. (C-D) HepG2 cells were transfected with siControl or siMDA-5. After 72 hours, the cells were infected with HAV at 200 GE/cell. Cell pellets and culture supernatants were harvested 24 hours after infection. Real-time qPCR (C) and ELISA (D) were performed to examine the production of CXCL10. Bar graphs represent the means ± s.e.m. (n = 3). Unpaired t-tests were performed. **P* < 0.05 and ****P* < 0.001 compared to siControl.

Supplementary Figure 4

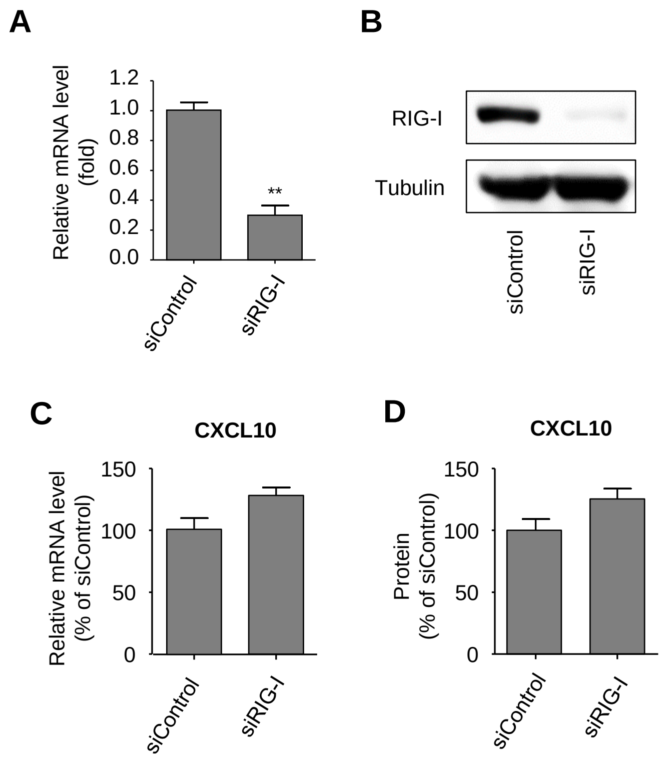

Supplementary Figure 4. RIG-I-independent production of CXCL10 in HAV-infected cells.
(A-B) HepG2 cells were transfected with siRNA targeting scrambled sequences (siControl) or RIG-I (siRIG-I). Efficient knock-down by siRIG-I was confirmed via real-time qPCR (A) and immunoblotting (B). Bar graphs represent the means ± s.e.m. (n = 3). Unpaired t-tests were performed. ***P* < 0.01 compared to siControl.
(C-D) HepG2 cells were transfected with siControl or siRIG-I. After 72 hours, the cells were infected with HAV at 200 GE/cell. Cell pellets and culture supernatants were harvested 24 hours after infection. Real-time qPCR (C) and ELISA (D) were performed to examine the production of CXCL10. Bar graphs represent the means ± s.e.m. (n = 3). Unpaired t-tests were performed. There was no statistical difference between two groups.
